# Interleukin-4 Receptor Alpha: From Innate to Adaptive Immunity in Murine Models of Cutaneous Leishmaniasis

**DOI:** 10.3389/fimmu.2017.01354

**Published:** 2017-11-10

**Authors:** Ramona Hurdayal, Frank Brombacher

**Affiliations:** ^1^Faculty of Health Sciences, Division of Immunology and South African Medical Research Council (SAMRC) Immunology of Infectious Diseases, Institute of Infectious Diseases and Molecular Medicine (IDM), University of Cape Town, Cape Town, South Africa; ^2^International Center for Genetic Engineering and Biotechnology (ICGEB), Cape Town, South Africa; ^3^Department of Molecular and Cell Biology Faculty of Science, University of Cape Town, Cape Town, South Africa

**Keywords:** interleukin-4 receptor alpha, interleukin-4/interleukin-13, murine cutaneous leishmaniasis, innate cells, adaptive cells

## Abstract

The interleukin (IL)-4 receptor alpha (IL-4Rα), ubiquitously expressed on both innate and adaptive immune cells, controls the signaling of archetypal type 2 immune regulators; IL-4 and IL-13, which elicit their signaling action by the type 1 IL-4Rα/gamma common and/or the type 2 IL-4Rα/IL-13Rα complexes. Global gene-deficient mouse models targeting IL-4, IL-13, or the IL-4Rα chain, followed by the development of conditional mice and generation of important cell-type-specific IL-4Rα-deficient mouse models, were indeed critical to gaining in-depth understanding of detrimental T helper (Th) 2 mechanisms in type 1-controlled diseases. A primary example being cutaneous leishmaniasis, which is caused by the protozoan parasite *Leishmania major*, among others. The disease is characterized by localized self-healing cutaneous lesions and necrosis for which, currently, not a single vaccine has made it to a stage that can be considered effective. The spectrum of human leishmaniasis belongs to the top 10 infectious diseases according to the World Health Organization. As such, 350 million humans are at risk of infection and disease, with an incidence of 1.5–2 million new cases being reported annually. A major aim of our research is to identify correlates of host protection and evasion, which may aid in vaccine design and therapeutic interventions. In this review, we focus on the immune-regulatory role of the IL-4Rα chain from innate immune responses to the development of beneficial type 1 and detrimental type 2 adaptive immune responses during cutaneous *Leishmania* infection. We discuss the cell-specific requirements of the IL-4Rα chain on crucial innate immune cells during *L. major* infection, including, IL-4Rα-responsive skin keratinocytes, macrophages, and neutrophils, as well as dendritic cells (DCs). The latter, contributing to one of the paradigm shifts with respect to the role of IL-4 instructing DCs *in vivo*, to promote Th1 responses against *L. major*. Finally, we extend these innate responses and mechanisms to control of adaptive immunity and the effect of IL-4Rα-responsiveness on T and B lymphocytes orchestrating the development of CD4^+^ Th1/Th2 and B effector 1/B effector 2 B cells in response to *L. major* infection in the murine host.

## Introduction

Human leishmaniasis, ranging from localized ulcerating lesions (cutaneous) to disseminated (mucocutaneous) and fatal infection (visceral), presents a global health concern, with over 12 million people currently infected and an additional 350 million humans at risk of infection and disease (1, 2). It is therefore not surprising that infection rates surpass 1.5 million new cases annually (3). Despite a concerted effort to develop a vaccine against the parasite, not a single candidate has been proven effective, and current therapeutic approaches are unable to achieve a sterile cure (2, 3). A thorough understanding of the mechanisms by which *Leishmania* spp. evade or exploit host immune mechanisms, to persist and establish disease, is paramount to identifying new and improved strategies for effective management of the disease. To address this, experimental models of cutaneous leishmaniasis (CL), which is the most common form of the disease, was established. In this model, disease is induced by infecting mice subcutaneously with *Leishmania major*. The *L. major* mouse model provided an excellent system for investigating the mechanisms underlying T helper (Th) 1 and Th2 cell differentiation relating to resistance and susceptibility to intracellular infection (4–6). This model, in global gene-deficient mice, established that the archetypal Th2 cytokines, interleukin-4 (IL-4) and interleukin-13 (IL-13), are susceptibility factors during *L. major* infection in BALB/c mice, counter-regulating a protective Th1 response, and induce their biological functions through a common receptor, the interleukin-4 receptor alpha (IL-4Rα) chain (7–9). However, IL-4Rα-deficient BALB/c mice remain susceptible to *L. major* infection in chronic stages (9, 10), indicating that IL-4/IL-13 may induce protective responses depending on which cell/s the IL-4/IL-13 ligand/s interact with during disease (11). Given the ubiquitous expression of the IL-4Rα signaling receptor on both innate and adaptive immune cells (12), cell-type-specific IL-4Rα-deficient mice were introduced to dissect the cell-specific roles of IL-4/IL-13 in CL. While these studies exemplified the role of IL-4Rα signaling on specific immune cells (10, 11, 13), it also questioned whether the Th1/Th2 paradigm of resistance/susceptibility to infection was in fact still relevant, considering that in certain disease settings, IL-4/IL-13 signaling essentially instructed a beneficial Th1 response (5, 11). Collectively, these reports highlight that the interplay between resistance and susceptibility to murine *L. major* infection involves a complex, dynamic interaction between the IL-4Rα chain and various innate and adaptive immune cells, with different clinical and immunological outcomes. In this review, we focus on the cell-specific requirements of the IL-4Rα chain signaling on crucial cells mediating innate and adaptive immunity to CL, relating primarily to *L. major* infection in mouse models.

## The IL-4Rα Chain: Common Receptor for IL-4 and IL-13 Signaling

### Interleukin-4

Interleukin-4 plays a critical role in initiating and regulating Th2-type immune responses (14). In mice, IL-4 is a 14–19 kDa glycoprotein localized on chromosome 11, together with the genes for IL-5 and IL-13. During the innate immune response, evidence suggests that early IL-4-producers include basophils (15, 16), mast cells (17), eosinophils (18), natural killer (NK) T cells (19, 20), and innate-like skin keratinocytes (21). T and B lymphocytes orchestrating adaptive immunity, specifically CD4^+^ Th2 cells (22), B effector 2 (Be2) B cells (23, 24), and γ/δ T cells (25), also secrete IL-4. Apart from regulating the differentiation of Th2 cells, IL-4 also controls immunoglobulin class switching in activated B cells, specifying human B cells to switch to the expression of IgE and IgG4 (26), while in mice, to IgE and IgG1, with the concomitant suppression of IgM, IgG2a, and IgG2b (27, 28). Moreover, alternatively activated macrophages are activated by IL-4 signaling through the IL-4Rα chain (29). Importantly, IL-4 inhibits inducible nitric oxide synthase (iNOS) expression thereby inhibiting IFN-γ-induced classically activated macrophages and induction of a type 1 response. As a whole, IL-4 counter-regulates the expression of IFN-γ (12) and increases the expression of MHC II molecules (30), co-stimulatory molecules CD80 and CD86 (31), and the IL-4 receptor (32). Reports have also indicated that dendritic cells (DCs) can respond to IL-4 *in vivo* and *in vitro* and become alternatively activated, in a manner similar to that described for alternatively activated macrophages, by upregulating multiple alternative activation markers such as mannose receptor and RELM-α (33). Moreover, although IL-4 has been shown to be the primary inducer of Th2 responses, studies have reported IL-4-independent Th2 differentiation, Th2 cytokine production, IL-4Rα signaling, and STAT6 regulation (34–41).

### Interleukin-13

Murine IL-13 is an immunoregulatory cytokine with a molecular weight of 10–14 kDa (42), also localized on chromosome 11, together with the genes for IL-4 and IL-5. Similar to IL-4, murine IL-13 also promotes upregulation of MHC II antigens, co-stimulatory (CD80/CD86) and adhesion molecules. However, unlike IL-4, murine IL-13 has been shown not to affect Th2 differentiation, B cell switching or upregulation of the low-affinity IgE receptor (CD23), likely due to the absence of a functional IL-13 receptor on those cells in mice. By contrast, human B lymphocytes do respond to IL-13 (34). Innate mast cells, basophils, DCs, NK cells, activated CD4^+^ Th2 cells, Be2 B cells, and NK T cells are IL-13-producers (34, 43–45). In fact, IL-13 is responsible for activating mast cells, modulating eosinophil function (42), and alternatively activating macrophages in conjunction with IL-4 (29). IL-13 also has immunosuppressive and anti-inflammatory effects on macrophages and monocytes, including suppression of pro-inflammatory cytokines and chemokines. In addition, nitric oxide (NO) production, along with antibody-mediated cytotoxicity, is inhibited by IL-13.

### The IL-4 and IL-13 Receptor Complexes

**Figure 1:**
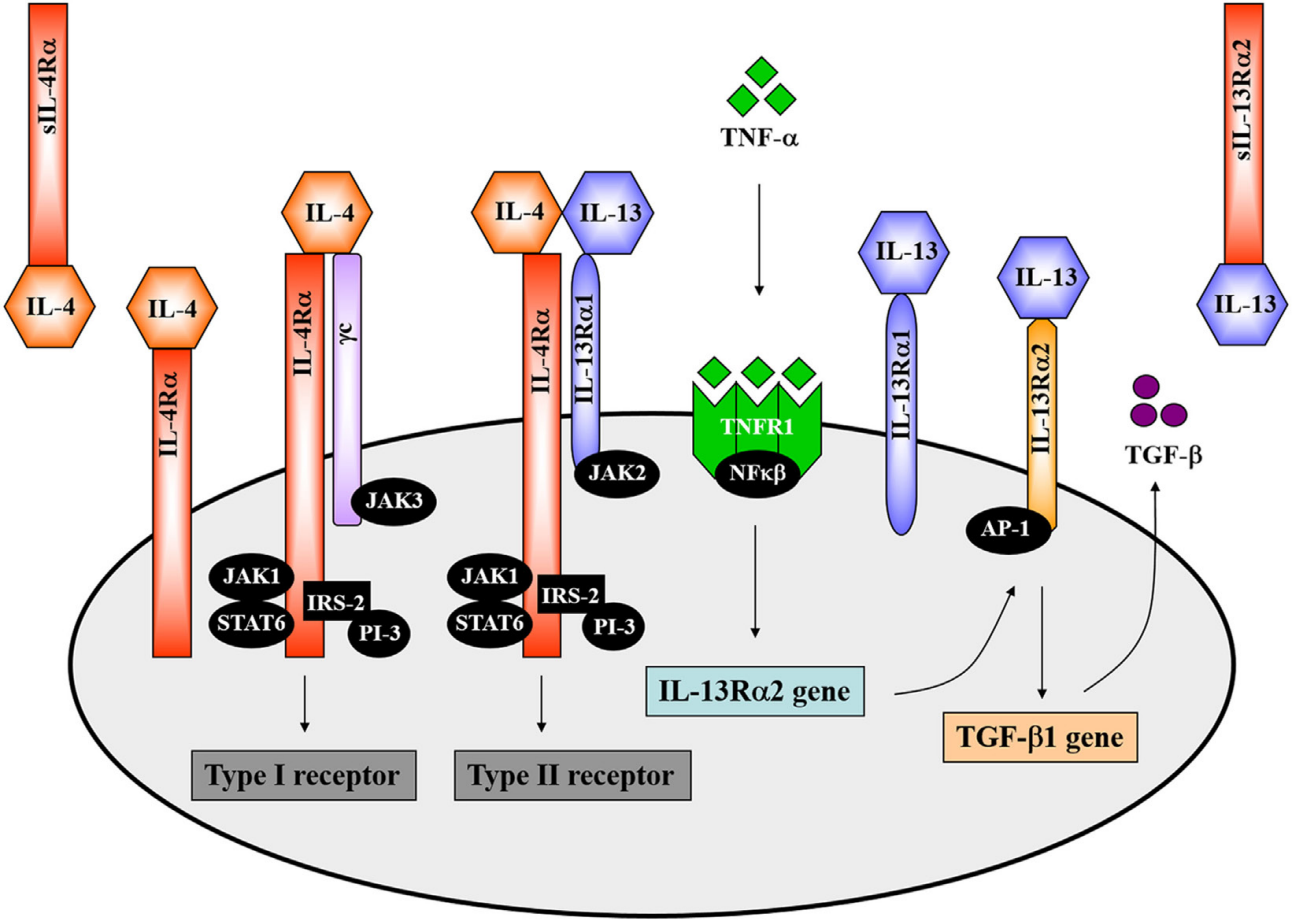
The interleukin-4 (IL-4) and interleukin-13 (IL-13) receptor complexes. IL-4 interacts with the interleukin-4 receptor alpha (IL-4Rα) chain in combination with either the gamma common (γc) chain to form the type 1 receptor or with the IL-13-binding receptor alpha 1 (IL-13Rα1) to form the type 2 receptor complex. The IL-4Rα chain signals *via* the JAK1/STAT6 pathway. Definitively, JAK3 associates with the γc chain and JAK2 with the IL-13Rα1. IL-13 interacts with the type II receptor complex (through IL-13Rα1) or with the α2 chain of the IL-13 receptor (IL-13Rα2). A signaling pathway for IL-13 *via* IL-13Rα2 has recently been identified. TNF-α induces upregulation of IL-13Rα2 expression. IL-13 then binds to the IL-13Rα2, which activates AP-1 to induce gene expression and secretion of soluble TGF-β. Illustration adapted and redrawn from previous publications (12, 34, 42, 52, 56).

The overlapping biological functions of IL-4 and IL-13 on certain cell-types could be partly attributed to the shared IL-4Rα component of natively distinct receptors (34). This theory was initially demonstrated in competitive studies in which treatment of mice with IL-4 antagonists or anti-IL-4Rα antibodies inhibited both IL-4- and IL-13-mediated responses (46–48). The IL-4Rα chain (CD124) is a 140 kDa heterodimeric complex, serving as a common monomer in both the type 1 and type 2 receptor complexes (Figure 1). It is ubiquitously expressed in fairly low numbers on hematopoietic and non-hematopoietic cells (12). IL-4Rα interacts with the gamma common (γc) chain to form the type 1 IL-4 receptor and interacts with the 65–70 kDa IL-13-binding receptor alpha 1 (IL-13Rα1) chain to form the type 2 IL-4/IL-13 receptor (Figure 1) (12). The former is also shared by the receptors for IL-2, IL-7, IL-9, and IL-15. IL-4 binds the IL-4Rα chain with high affinity. By contrast, IL-13 binds the IL-13Rα1 chain with low affinity. However, when paired with the IL-4Rα chain, IL-13 binds the IL-13Rα1 chain with high affinity forming an active signaling unit (49). Expression of IL-13Rα1 is absent on human or murine T cells but constitutively expressed on B cells, epithelial cells and monocytes in both mice and humans (50, 51). In comparison, IL-13 shows a higher binding affinity for the α2 chain of the IL-13 receptor (IL-13Rα2), which is a 55–60 kDa protein (Figure 1). IL-13Rα2 was originally considered a decoy receptor for IL-13, devoid of signal transduction, since its short cytoplasmic domain was not reported to contain any binding motifs for signaling molecules (52). However, well-designed reports have demonstrated a signaling pathway for IL-13 through the IL-13Rα2 chain, which induces the production of TGF-β1 and mediates fibrosis (53). In addition to cell-surface localization, soluble forms of both IL-4Rα and IL-13Rα2 exist (Figure 1), which are capable of binding IL-4 and IL-13 with high affinity as non-signaling monomers. In doing so, the soluble receptors can act as competitive inhibitors of both IL-4 and IL-13 and modulate their effector responses (54, 55).

### Mechanisms of IL-4 and IL-13 Signaling through the IL-4Rα Chain

Both IL-4 and IL-13 signal transduction *via* the IL-4Rα chain involves activation of the Janus-family kinases (JAK). JAK1 interacts with the IL-4Rα chain whereas JAK3 interacts with the γc chain and JAK2 with the IL-13Rα1 (Figure 1) (12, 57, 58). IL-4 engagement with the IL-4Rα chain results in tyrosine phosphorylation of the IL-4Rα chain itself as well as phosphorylation of STAT6 and insulin receptor substrate 2 (IRS-2) by JAKs, which then associates with the phosphoinositol-3 kinase (PI-3) (Figure 1) (12, 34). STAT6-deficient mice presented impaired IL-13-mediated functions, which confirmed that IL-13 also uses the JAK/STAT6 pathway for signal transduction (Figure 1) (34, 59). IL-13 signaling through the IL-13Rα2 requires initial engagement of the IL-13Rα1/IL-4Rα complexes in conjunction with TNF-α signaling, which increases surface expression of IL-13Rα2. IL-13 then binds to the IL-13Rα2 and, through activation of the transcription factor AP-1, drives secretion of TGF-β (Figure 1) (52, 53).

## Cutaneous Leishmanisis

### Cell-Mediated Host Immune Responses to *L. major*

**Figure 2:**
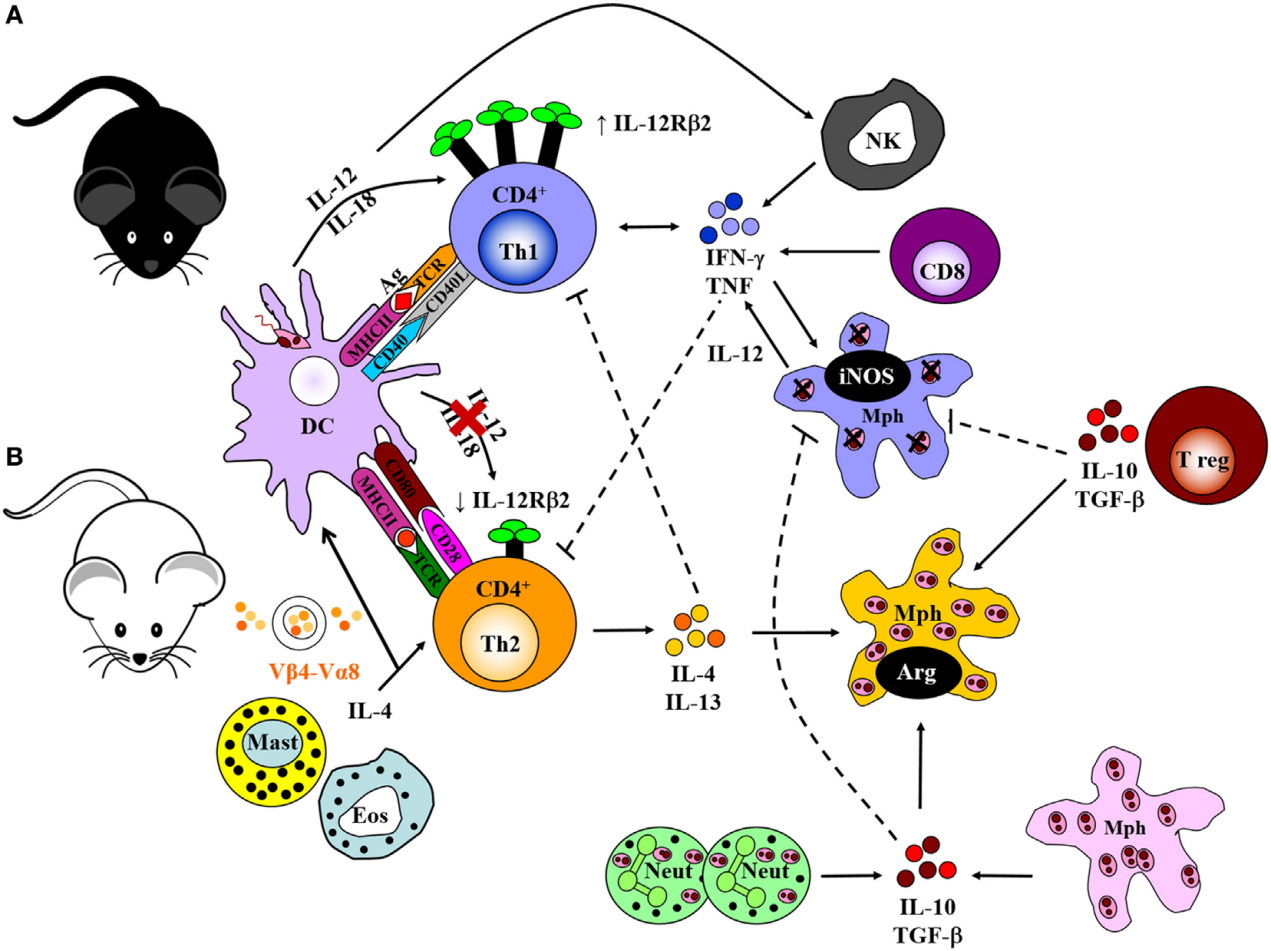
Cell-mediated immune response to *Leishmania major* in resistant and susceptible mice. **(A)** Upon interaction with *L. major* parasites, activated dendritic cells (DCs) process and present parasite antigen and produce IL-12 and IL-18, which interact with the IL-12 receptor β2-chain (IL-12Rβ2) in activated Th1 cells. Together, a dominant Th1 response is generated by the production of IFN-γ and TNF-α. These cytokines upregulate the expression of inducible nitric oxide synthase (iNOS) and activate infected macrophages for intracellular killing. CD8^+^ T cells and natural killer (NK) cells control infection by producing IFN-γ and effector killing by macrophages leading to parasite control as observed in C57BL/6 mice. **(B)** By contrast, parasite persistence in BALB/c mice is due to the failure of an IL-12-dependent redirection of the early Th2 response. This may be due to continued expression of early interleukin-4 (IL-4) from Vβ4^+^Vα8^+^ CD4^+^ T cells, mast cells, and eosinophils at the inoculation site and draining lymph nodes. This delays secretion of IL-12, or loss of responsiveness to IL-12 due to reduced expression of the IL-12Rβ2 chain on activated Th2 cells. IL-4 and IL-13 suppress Th1 responses and inhibit intracellular killing by macrophages. This leads to arginase production by macrophages that favor parasite growth. Sustained neutrophil recruitment to the site of infection and regulatory T cells may promote susceptibility and inhibit Th1 responses by the production of IL-10 and TGF-β that inhibit activation of macrophages. Solid lines represent activation, and broken lines represent inhibition. Illustration drawn from previous publications (6, 75, 76).

The *Leishmania* parasite has a complex digenetic life cycle alternating between two distinct stages; the promastigote form found in the female *Phlebotomus* sandfly vector and the amastigote form replicating within phagolysosomes of host macrophages and DCs. Ironically, macrophages (and DCs) being the primary immune cells involved in the eradication of *Leishmania* in a mammalian host, are the preferred host cells of the parasite (3, 11, 60, 61). Early pioneering studies focused on the modulation of cytokine signaling as a means to alter immune cell activation and Th cell differentiation. These studies were conducted in an attempt to understand the mechanisms by which *Leishmania* are able to survive and flourish in these hostile environments while also capitalizing on host defense mechanisms to favor the establishment of disease. These mouse models, involving experimental infection with *L. major*, established the Th1/Th2 paradigm of resistance/susceptibility to intracellular infection (5, 6). Upon infection with *L. major*, genetically resistant C57BL/6 mice develop a healing phenotype associated with an IL-12-driven protective Th1/type 1 immune response, upregulation of IFN-γ and classical activation of macrophages for subsequent killing of intracellular parasites by effector NO production (Figure 2) (13, 62–66). IL-18 augments IL-12 activity, both of which interact with the IL-12 receptor β2-chain (IL-12Rβ2) in activated Th1 cells (67) and protects against *L. major* infections (68). CD8^+^ T cells and NK cells enhance this protective response by secreting IFN-γ that activates macrophages for parasite clearance. By contrast, the lack of healing in genetically susceptible BALB/c mice is associated with a Th2/type 2 response characterized by the secretion of IL-4, IL-5, IL-9, IL-10, and IL-13, high type 2 anti-*Leishmania* antibody titers, and alternative activation of macrophages, which altogether promotes parasite survival and growth (Figure 2) (7, 8, 69–71). BALB/c mice present with lower levels of IL-12. Evidence suggests that this might be due to sustained IL-4 expression by Vβ4^+^Vα8^+^ CD4^+^ T cells, mast cells and eosinophils at the inoculation site and draining lymph node (LN) (Figure 2), which delays secretion of IL-12. Alternatively, reduced expression of the IL-12Rβ2 chain on activated Th2 cells lead to a loss in responsiveness to IL-12. In addition, maintenance of inflammatory neutrophils at the site of parasite inoculation (72) and regulatory T cells (Tregs), constituting a source of IL-10, favors the persistence of parasites in leishmanial skin lesions (Figure 2) (73, 74).

The early IL-4 response to *L. major* is confined to CD4^+^ T cells expressing a Vβ4^+^Vα8^+^ T cell receptor that recognizes the *Leishmania* antigen LACK (*Leishmania* homolog of receptors for activated C kinase) (14, 77), in addition to mast cells and eosinophils at the site of infection and draining LN. Both BALB/c and C57BL/6 mice secrete IL-4 early after infection; however, production of IL-4 is sustained in susceptible BALB/c mice and transient in resistant C57BL/6 mice (78). The resistant mice redirect the early Th2 response in an IL-12-dependent mechanism toward a beneficial Th1 response, whereas in susceptible mice, the Th2 response persists and dominates the disease outcome by suppressing effector mechanisms essential for parasite killing (6, 79).

### IL-4/IL-13 and IL-4Rα Signaling in CL

**Figure 3:**
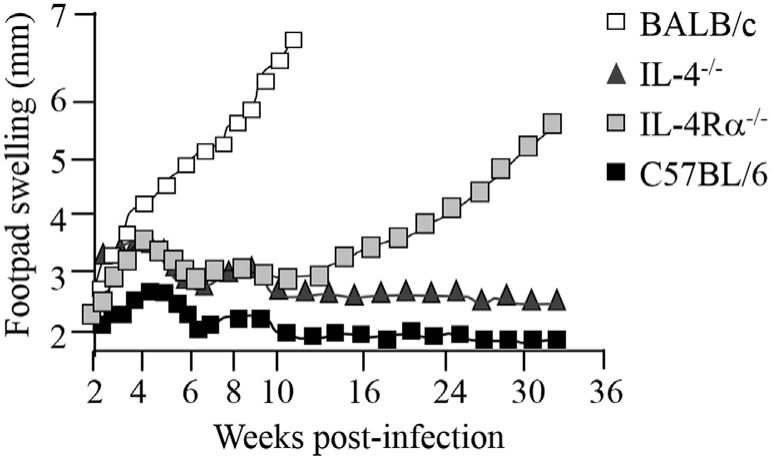
Disease progression in *Leishmania major*-infected-IL-4^−/−^ and IL-4Rα^−/−^ mice. BALB/c mice deficient for interleukin-4 (IL-4) and interleukin-4 receptor alpha (IL-4Rα) were infected with *L. major* LV39 strain and footpad swelling monitored weekly as an indication of disease progression. Illustration prepared according to published reports (7, 9–11).

Since both IL-4 and IL-13 share a common signaling pathway through the IL-4Rα chain, collectively modulating type 2 immunity, IL-4Rα-mediated mechanisms became our primary research interest, in cutaneous and visceral leishmaniasis (7–9, 80) and extending to acute schistosomiasis (81–83), nematode infections (84–86), and allergic airway disease (87, 88). Deletion of the IL-4Rα component impairs downstream signaling of both IL-4 and IL-13 responses *via* transcription factor STAT6 (Figure 1). This has been established in animal studies during *L. major* infection in which susceptible BALB/c mice deficient for IL-4 (7), IL-13 (8), IL-4Rα (9, 36), or STAT6 (89) were able to control disease progression. Initial control of *L. major* during the acute phase of infection in IL-4^−/−^-deficient mice (7) and IL-4Rα^−/−^-deficient (9) BALB/c mice is equivalent, with both strains of mice showing reduced footpad swelling (Figure 3), parasite loads and type 1 antibody responses (9). However, in contrast to IL-4^−/−^ mice, IL-4Rα^−/−^ mice developed progressive disease and necrotic footpad lesions during the chronic phase of infection (Figure 3). Thus, the absence of IL-13-mediated functions in IL-4Rα^−/−^ mice implicated IL-13 as a susceptibility factor in chronic *L. major* infection (9). This was confirmed in IL-13 transgenic C57BL/6 mice, which developed a susceptible phenotype to acute leishmaniasis with impaired IL-12 and IFN-γ production, whereas IL-13-deficient BALB/c mice remained comparatively resistant (8, 90). This was attributed to IL-13 promoting susceptibility by activating alternative macrophages and suppressing secretion of NO, IL-12, and/or IL-18 (8, 90).

The role of IL-4 and IL-4Rα in *Leishmania* infection in mice is, however, controversial since contradictory reports have suggested that although IL-4 is important, it is not the sole mediator of susceptibility in BALB/c mice. While Kopf et al. and Mohrs et al. demonstrated that IL-4^−/−^ and IL-4Rα^−/−^ mice were able to control CL (7, 9) (Figure 3), Noben-Trauth et al. showed that IL-4^−/−^- and IL-4Rα^−/−^-deficient BALB/c mice remained susceptible to disease and could not contain parasites at the site of infection (37, 91). This discrepancy between the two studies and in general, the outcome of *L. major* infection, may be attributed to a variation of experimental factors, such as the parasite sub-strain used, level of parasite virulence and the embryonic stem cells used to create the knockout mice. Of recent interest, the complex interplay between commensal microbiota in host animals and their vicinity may also be a contributing factor (92). Important to note, despite the absence of IL-4 or IL-4Rα, Th2-cell development and Th2-related cytokines were significantly promoted, although in different Th1/Th2 ratios (7, 37, 91). Subsequently, studies sought to follow IL-4 expression and Th cell development *in vivo* in *L. major*-infected IL-4^−/−^ and IL-4Rα^−/−^ BALB/c mice (36, 38, 93). The results revealed that, despite a clear absence of IL-4/IL-13-mediated functions in IL-4^−/−^ and IL-4Rα-deficient mice, unimpaired Th2 polarization and IL-4-producing CD4^+^ T cells as well as other Th2-related cytokines were still present (7, 34, 36, 38). Accordingly, these pioneering studies confirmed that both IL-4-dependent and IL-4-independent factors contribute to the susceptibility phenotype in *L. major*-infected BALB/c mice. Together, these findings contradicted the idea that IL-4 is the sole regulator of susceptibility to *L. major* infection. The body of literature alternatively suggested that the combined action of IL-4/IL-13 heightens susceptibility to *L. major*, nonetheless, both cytokines may in fact act independently of each other to induce a non-healing response. More importantly, Th2 and type 2 immune responses may be induced independently of IL-4 and IL-13. Thus, at this stage 15 years ago, a definitive role for IL-4/IL-13 in progression of CL remained questionable.

Expression of the IL-4Rα chain reflects the pleiotropic nature of IL-4/IL-13 biology, as this receptor complex is endogenously expressed upon a diverse range of innate and adaptive immune cells in the host. In global gene-deficient mice, the target gene is deficient on all hematopoietic cells. Thus, IL-4- and IL-13-mediated functions at a cellular level remain uncharacterized. Therefore, it is possible that the range of target cells interacting with IL-4 and IL-13, and their order of hierarchical effector function or importance in the host, may account for differential IL-4/IL-13-mediated mechanisms during cutaneous *Leishmania* infection (4, 5). The emerging principles of innate instruction of adaptive immunity further exemplify that IL-4Rα signaling on innate cells may contribute specific functions that shape the adaptive immune response and outcome of infection.

## IL-4Rα Signaling on Specific Innate Immune Cells in CL in Mice

### Cre/*lox*P Targeting to Generate Cell-Specific IL-4Rα-Deficient Mice

**Figure 4:**
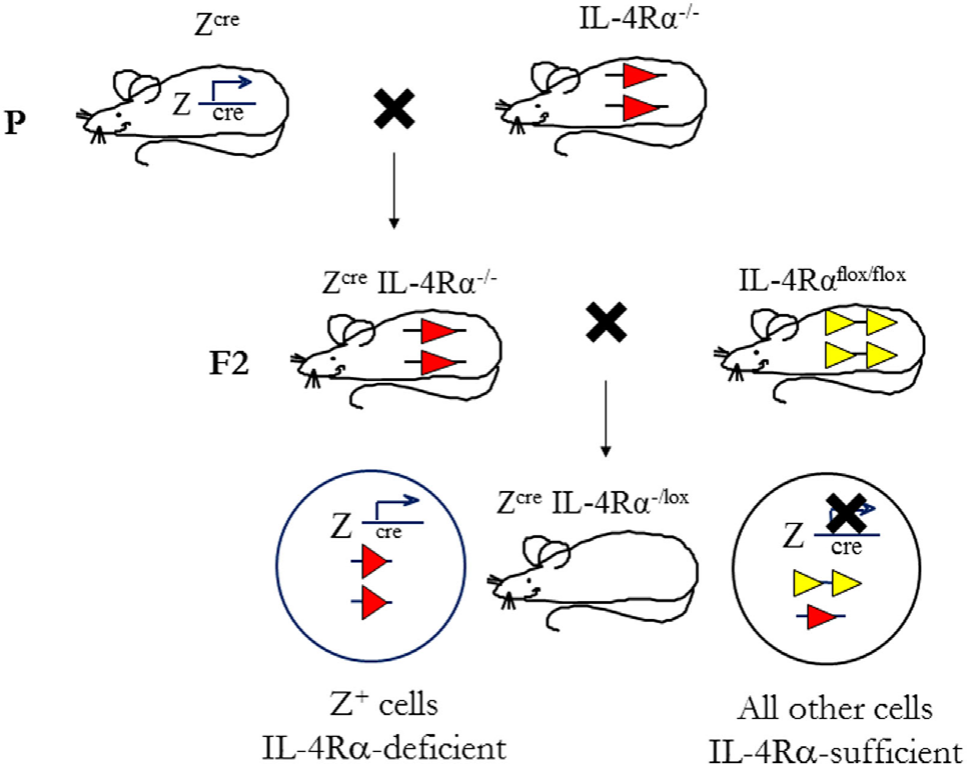
Mouse breeding strategy to create cell-specific interleukin-4 receptor alpha (IL-4Rα)-deficient mice. IL-4Rα^flox/flox^ BALB/c mice were intercrossed with transgenic mice expressing Cre-recombinase under control of a cell-specific promoter (Z^cre^) and IL-4Rα^−/−^ BALB/c mice to generate Z^cre^IL-4Rα^−/lox^ mice. The “loxed” IL-4Rα allele, yellow arrows; deleted allele, red arrows. Illustration adapted and redrawn from previous publications (10, 11, 82).

To determine the cell-specific requirements for the IL-4Rα and its ligands, in type 1 and type 2-controlled diseases, we established a second generation of knockout mice using conditional IL-4Rα-deficient mice, thus creating novel mice with a cell-specific deletion of the *il4r*α gene. This was achieved by the bacteriophage-derived Cre/*lox*P genetic recombination system under control of specific loci (Figure 4). In this system, cyclization recombinase (Cre) inserted downstream of a cell-specific promoter recognizes a pair of *lox*P sequences flanking the gene of interest (specifically, Exon 7 to Exon 9 of IL-4Rα). Cre-recombinase removes the intervening DNA by bringing the two *lox*P sites together (94). Transgenic Cre mice (Z^cre^) are backcrossed to BALB/c or C57BL/6 for nine generations, then intercrossed with global IL-4Rα (IL-4Rα^−/−^) BALB/c (9) mice to generate Z^cre^IL-4Rα^−/−^ BALB/c mice. Littermate mice are then subsequently intercrossed with floxed IL-4Rα (IL-4Rα^flox/flox^) BALB/c mice (Exon 6 to Exon 8 flanked by *lox*P) (82) to yield cell-specific IL-4Rα-deficient mice (or Z^cre^IL-4Rα^−/lox^) (10, 82, 84). With this strategy, we increase the efficiency of Cre-recombination by reducing the LoxP substrate for Cre-recombinase by 50%, thereby avoiding early aberrant non-Mendelian Cre recombination (10, 82).

### IL-4Rα-Responsive Innate-Like Skin Keratinocytes in Murine CL

**Figure 5:**
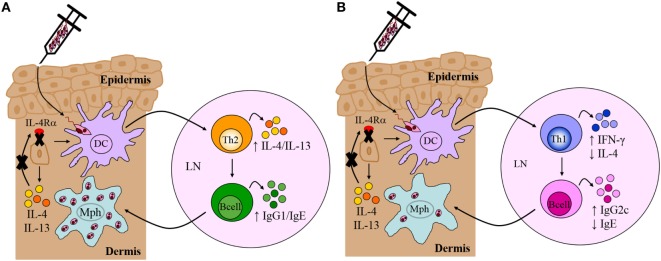
Immune response in *Leishmania major*-infected BALB/c and C57BL/6 mice deficient for interleukin-4 receptor alpha (IL-4Rα)-responsive keratinocytes. **(A)** Th2/Type 2-mediated susceptibility in BALB/c mice deficient for IL-4Rα-responsive keratinocytes. KRT14^cre^IL-4Rα^−/lox^ BALB/c mice, generated by cre/*lox*P targeting, developed non-healing disease to experimental *L. major* LV39 and IL81 infection in the footpad, characterized by substantial interleukin-4 (IL-4)/interleukin-13 (IL-13) production and IgG1/IgE production, similar to littermate control BALB/c mice. Model prepared using information from submitted manuscript. **(B)** Th1/Type 1-mediated protective immunity in C57BL/6 mice deficient for IL-4Rα-responsive keratinocytes. Similar to control C57BL/6 mice, KRT14^cre^IL-4Rα^−/lox^ C57BL/6 mice, generated by cre/*lox*P targeting, controlled lesion development upon *L. major* LV39 and IL81 infection in the footpad or ear dermis, characterized by strong IFN-γ and IgG2c production and concomitantly reduced IL-4 and IgE (103). DC, dendritic cells; Mph, macrophage; LN, lymph node; Th1, T helper 1; Th2, T helper 2.

In CL, the skin represents the site of primary infection between the sandfly, parasites and the mammalian host. It is therefore probable that this immunologic organ may provide decisive signals for the development of a Th response early after infection. In addition to 20 billion T cells (95), the skin also contains innate and innate-like cells such as keratinocytes, melanocytes, and Langerhans cells in the epidermis, and dermal DCs, plasmacytoid DCs, phagocytes as well as lymphoid cells in the dermis (96). Keratinocytes are the most abundant cells in the epidermal layer (~90%) capable of differentiating harmful pathogens from harmless commensal organisms, and directing an appropriate immune response against it. The latter is due to their expression of distinct pattern recognition receptors (PRRs) that recognize signature pathogen-specific molecular patterns. Biological activities of keratinocytes range from secretion of antimicrobial peptides to recruitment of host immune cells and notably, modulation of cytokine production (96–98). An immunomodulatory role for keratinocytes during *L. major* infection became evident when it was found that these cells secreted an array of cytokines, such as IL-12, TNF-α, IL-1β, and IL-6, within the first few hours after infection. The release of keratinocyte-specific inflammatory cytokines following *Leishmania* exposure implies the involvement of Toll-like receptor (TLR) activation on these cells since TLR recognition is often associated with the production of pro-inflammatory cytokines (99). Of interest however, besides the release of pro-inflammatory cytokines, IL-4 was also found to be produced by keratinocytes of both BALB/c and C57BL/6 mice (21). While the general consensus is that IL-4 acts as a canonical Th2 cytokine to induce a detrimental Th2/type 2 response, there have been studies demonstrating that early production of IL-4 at the site of infection may essentially drive a beneficial Th1 response, under the instruction of DCs secreting IL-12 (11, 100, 101). Importantly, direct uptake of *L. major* parasites by keratinocytes seems unlikely because of the subcutaneous infection and because keratinocytes have been reported not to take up *L. major in vitro* (21). Nevertheless, these reports suggested that keratinocytes may be the source of the early IL-4 that instructs DCs to drive a protective Th1/type 1 response. This is supported by the fact that keratinocytes express surface IL-4Rα predisposing the cells to possible autocrine stimulation by keratinocyte-derived IL-4 and IL-13, which may also influence DCs in a paracrine manner. Of note, Ehrchen et al. (21) did not identify strain-specific differences in the expression of IL-13 in the skin or specifically by keratinocytes. These cells can, however, secrete and signal IL-13 (97, 102). We therefore investigated if the autocrine action of IL-4 and IL-13, signaling on keratinocytes *via* the IL-4Rα complex, contributes to type 2 responses during CL. To address this, we generated keratinocyte-specific-IL-4Rα deficient (KRT14^cre^IL-4Rα^−/lox^) mice on a BALB/c background using the cre/*lox*P system (Figure 4) under control of the keratinocyte 14 (*krt14*) locus. Considering that the strain of *Leishmania* initiating infection has a relevant influence on T cell-priming during infection, we incorporated experimental CL with *L. major* LV39 and IL81 strains by subcutaneous injection in the footpad. Surprisingly, despite minor immunological changes, KRT14^cre^IL-4Rα^−/lox^ mice on the BALB/c background developed non-healing disease similar to the littermate control mice following infection in the footpad. Moreover, the default Th2/type 2-driven cellular and humoral immune response characteristic of BALB/c mice, developed independently of IL-4Rα-responsive keratinocytes following *L. major* LV39 and IL81 infection in the footpad (Figure 5A) (submitted manuscript). Overall, our data and the reports above suggests that cytokine and chemokine-secreting skin keratinocytes might be involved in amplifying the *L. major*-induced inflammatory tissue signal by mobilizing leukocytes to the infection site following recognition of *L. major* parasites by keratinocyte-specific PRRs.

In contrast to our original hypothesis, this suggested that IL-4Rα-responsive keratinocytes may not play a role in regulating the default Th2 polarized response during *L. major* infection in BALB/c mice. Nevertheless, given that Ehrchen et al. (21) reported a higher secretion of keratinocyte-derived IL-4 in C57BL/6 mice, as opposed to BALB/c, this suggested that the absence of IL-4 on keratinocytes in C57BL/6 mice might have a pronounced effect on Th1 polarization. As above and in Figure 4, the cre/*lox*P system was therefore used to generate KRT14^cre^IL-4Rα^−/lox^ mice on a C57BL/6 background. The use of both *L. major* LV39 and IL81 strains, injected subcutaneously in the footpad and ear, once again revealed unexpected results. In the absence of IL-4Rα-responsive keratinocytes, C57BL/6 mice controlled the development of inflammatory lesions upon infection with *L. major* LV39 and IL-81, which correlated with reduced parasite burdens and the expansion of Th1/type 1 cellular and humoral immune responses (Figure 5B) (103). Collectively, the data obtained in both KRT14^cre^IL-4Rα^−/lox^ BALB/c and C57BL/6 mice mitigate an autocrine role for IL-4/IL-13 signaling on keratinocytes in the development of a non-healing Th2/type 2 or protective Th1/type 1 immune response, respectively, following experimental infection with *L. major* LV39 and IL-81 in mice.

### IL-4Rα-Responsive Macrophages and Neutrophils in Murine CL

Following *Leishmania* infection in the skin, innate macrophages, neutrophils and DCs are recruited to the site of inoculation, which can become infected and as a result, have specific and important roles in shaping CD4^+^ Th cell-dependent immune responses to infection. Considering that each of these cell-types express the surface IL-4Rα complex, we investigated cell-specific requirements for the IL-4Rα and its ligands on macrophages, neutrophils, and DCs. Studies have demonstrated that *Leishmania*-derived molecules activate TLRs, such as TLR2, TLR4, and TLR9, on these professional phagocytes to initiate the innate response. However, the consequences of such activation are complex and depend on the nature of the TLR, the cell type, the parasite species, the timing in which these events occur and the cytokine milieu surrounding the infected cells (104). Concerning macrophages, it is now well-accepted that these cells can be activated by different stimuli, with IFN-γ leading to classically activated macrophages, while signaling of IL-4/IL-13, *via* the IL-4Rα complex, results in alternative macrophage activation. The former mediates secretion of pro-inflammatory cytokines and killing of intracellular *Leishmania*, while the latter favors growth and survival of the parasites. Importantly, iNOS-mediated NO production is counter-regulated by IL-4Rα-dependent mechanisms through depletion of l-arginine, the substrate utilized by iNOS, leading to arginase I-expressing alternatively activated macrophages. Therefore, we postulated that the absence of IL-4Rα-responsive macrophages at the site of infection would induce signals that lead to a polarized protective Th1 response due to the absence of alternatively activated macrophages. Accordingly, LysM^cre^IL-4Rα^−/lox^ mice on a susceptible BALB/c genetic background were generated, by cre/*lox*P targeting under control of the lysosome (*lysm*) locus (Figure 4), showing selective deficiency of IL-4Rα on macrophages and neutrophils.

**Figure 6:**
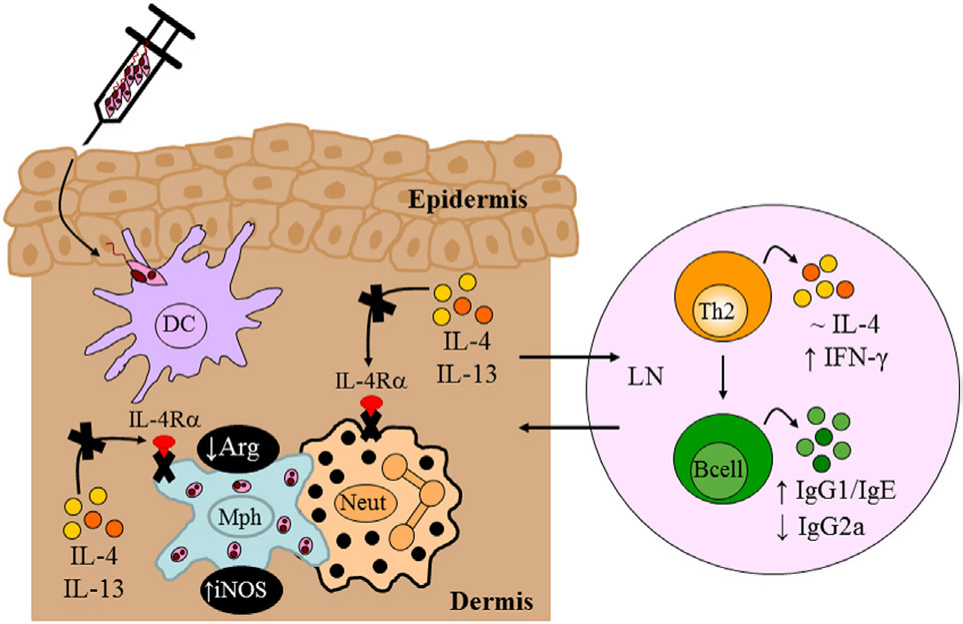
Immune response in BALB/c mice deficient for interleukin-4 receptor alpha (IL-4Rα)-responsive macrophages/neutrophils. Despite delayed disease progression in the footpads of *L. major* LV39-infected LysM^cre^IL-4Rα^−/lox^ mice, unimpaired Th2/type II responses and reduced IgG2a were detected, similar to control BALB/c mice. The absence of IL-4Rα-responsive macrophages led to increased inducible nitric oxide synthase (iNOS) and reduced arginase, reflecting a shift to classically-activated macrophages likely as a consequence of increased IFN-γ compared to littermate control BALB/c mice. Illustration prepared from Hölscher et al. (13). DC, dendritic cells; Mph, macrophage; LN, lymph node; Th2, T helper 2.

In the context of *Leishmania* infection, neutrophils rapidly recruited to the site of inoculation (105) mediate persistence of parasites in skin lesions (72, 106). By contrast, it has also been shown that neutrophils mediate killing of intracellular *Leishmania* by neutrophil extracellular traps (107) or *via* activation of killing effector functions in macrophages through recruitment of TLR4 by neutrophil elastase (104, 108). However, the importance of type 2 signaling on macrophages and neutrophils during the early stages of *L. major* infection was undefined. The LysM^cre^IL-4Rα^−/lox^ BALB/c strain therefore provided an attractive model to interrogate the combined contribution of Th2/type 2 responses *via* the IL-4Rα on macrophages and neutrophils *in vivo* in progression of CL. Hölscher et al. (13) revealed that in the absence of IL-4Rα on macrophages and neutrophils, BALB/c showed a significant control of disease progression up to 13 weeks after infection with *L. major*, despite sufficient Th2 and type 2 immune responses similar to littermate controls (Figure 6). Delayed disease progression in *L. major*-infected LysM^cre^IL-4Rα^−/lox^ BALB/c mice was attributed to inhibition of alternative activation of macrophages and improved macrophage leishmanicidal activities due to NO production by classically activated macrophages (Figure 6). However, following 13 weeks of infection, LysM^cre^IL-4Rα^−/lox^ mice developed fulminant CL with increasing footpad swelling, accompanied by ulceration and necrosis concomitant with elevated parasite burdens requiring termination of the experiment by week 18. The action of macrophages and neutrophils appear to complement each other as both population of cells are rapidly recruited to the site of infection and capable of phagocytosing *Leishmania* parasites following TLR stimulation. Accordingly, TLR4 was shown to be required for efficient leishmanicidal activity as the absence thereof led to increased arginase-derived urea and reduced formation of NO, probably *via* the activity of iNOS. The latter requires IL-12 secretion by antigen-presenting cells (APCs), and this cytokine can be activated by TLR9 (109). Thus, once could speculate that enhanced TLR4/TLR9 activation might have contributed to early control of disease in LysM^cre^IL-4Rα^−/lox^ mice. Altogether, our study showed that IL-4Rα-mediated effects on macrophages and neutrophils seem to be involved in the development of early disease progression after *L. major* infection. The authors postulated that the transient effect of IL-4Rα-deficiency on macrophages/neutrophils after *L. major* infection may be that production of IL-4 and/or immunosuppressive cytokines by CD4^+^ T cells abolished the initial protective immune responses observed. Concurrently, an alternate hypothesis on IL-4Rα-dependent protective mechanisms emerged. Macrophage-derived IL-12 production was shown to be inhibited upon engagement of the IL-4Rα complex (82). Thus, Hölscher et al. (13) postulated that IL-4-mediated instruction of DCs may produce IL-12 (100), which indeed has been shown to promote resistance in *L. major*-infected BALB/c mice.

### IL-4Rα-Responsive DCs in Murine CL

Evidence from *in vitro* and *in vivo* studies began to contradict the detrimental role of IL-4 in susceptibility to *L. major* infection and the following observations proved critical: first, recombinant IL-4 was shown to promote IL-12 production by bone marrow-derived dendritic cells (BMDCs) upon stimulation with specific ligands; second, IL-4, not IL-13, enhanced the production of IL-12 *via* signaling through the type 1 IL-4R complex; third, exogenous IL-4-administered *in vivo* during the period of DC activation in a murine model of *L. major* infection upregulated DC-derived *il-12* transcripts leading to a protective Th1 response and healing in otherwise susceptible BALB/c mice, and fourth, administration of exogenous IL-4 *in vivo* during the period of T cell priming resulted in the default Th2 pathway and progressive disease in *L. major*-infected BALB/c mice. A follow-up study demonstrated that IL-4-mediated instruction was limited to DCs, excluding other APCs, and the mechanism responsible was inhibition of IL-10 by IL-4, leading to protective IL-12-driven Th1 responses (110). Altogether, the reports alluded to above portrayed IL-4 signaling on DCs as a double-edged sword in murine CL, emphasizing that the release of DC-derived IL-12 and the outcome of *L. major* infection in BALB/c mice depends on the timing of the IL-4Rα chain interacting with target cells. DCs are those innate cells that most acutely reflect innate control of adaptive immunity (111) as these sentinels of the immune system transition innate to adaptive immune responses owing to their proficient antigen-presenting function and migratory capacity (112).

**Figure 7:**
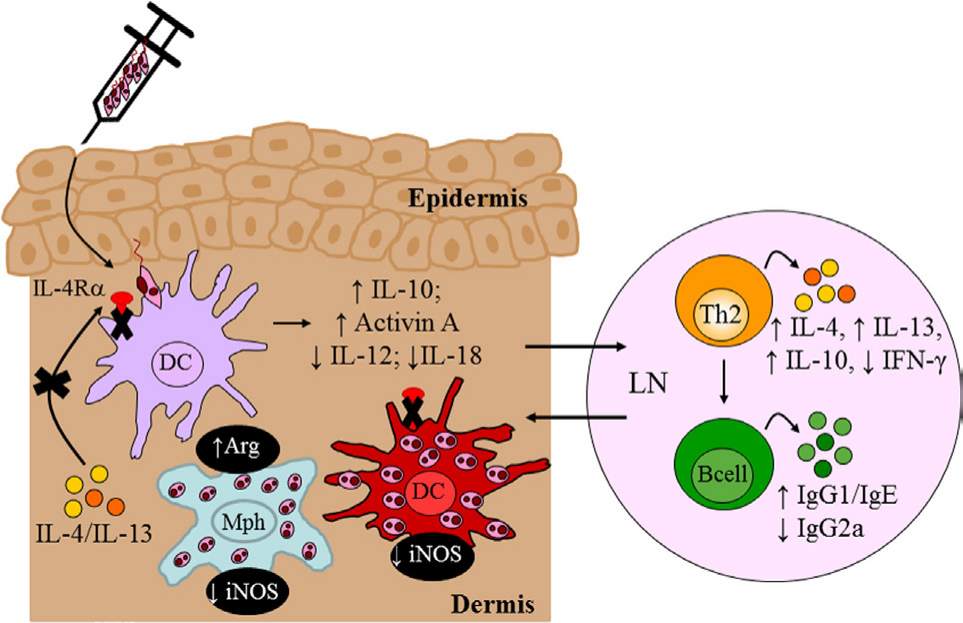
Immune response in BALB/c mice deficient for interleukin-4 receptor alpha (IL-4Rα)-responsive DCs. Absence of interleukin-4 (IL-4)/interleukin-13 (IL-13) signaling on DCs led to hypersusceptibility in BALB/c mice upon *Leishmania major* LV39 and IL81 infection in the footpad, characterized by a shift to Th2/type II immune responses and alternatively activated macrophages stronger than that usually observed in littermate BALB/c mice. Expression of inducible nitric oxide synthase (iNOS) in DCs was also dramatically reduced with increased arginase (Arg) compared to littermate mice providing a safe haven for *L. major* growth, survival, and dissemination to peripheral organs. Illustration prepared from Hurdayal et al. (11). DC, dendritic cells; Mph, macrophage; LN, lymph node; Th2, T helper 2.

To investigate a role for IL-4Rα-responsive DCs in resistance to *L. major* infection, we generated CD11c^cre^IL-4Rα^−/lox^ BALB/c mice, in which dendritic-cell specific deletion of the *il4r*α gene was achieved by the Cre/*lox*P system under control of the *cd11c* locus (Figure 4). Infection studies with *L. major* LV39 and IL81 in the footpad revealed that IL-4-mediated instruction of DCs occurs *in vivo* with biological quantities of IL-4 acting on DCs to promote protective immunity. In the absence of IL-4Rα-responsive DCs, BALB/c mice became hypersusceptible to disease, when compared with littermate control mice, showing exacerbated lesion development and earlier tissue necrosis. This correlated with uncontrolled parasite replication at the infection site and a shift to Th2/type 2 cellular and humoral immune responses (Figure 7). Notably, CD11c^cre^IL-4Rα^−/lox^ BALB/c mice displayed a striking increase in parasite dissemination from the site of infection to the draining LN and peripheral organs, including the brain of infected animals. Collectively, the data appeared to suggest that impaired NO-mediated killing effector functions in IL-4Rα-unresponsive DCs (and macrophages) facilitated a safe haven for parasite multiplication and dissemination of parasites. Accordingly, secretion of IL-12 by IL-4Rα-deficient DCs was severely impaired whereas IL-10 production was increased thereby confirming the mechanism behind impaired DC instruction *in vivo*. Interestingly, efficient TLR ligation on DCs disposes a potent negative signal for Th2 cell development (113). Thus, an alternate mechanism for the heightened susceptibility in CD11c^cre^IL-4Rα^−/lox^ BALB/c might involve impaired TLR-mediated activation of DCs. In agreement, induction of BMDC-IL-12 following *L. major* infection was shown to be dependent on TLR9 activation (114). This might suggest a combined involvement of IL-4 and the TLR pathway in activating DC-derived IL-12 in this protective anti-*L. major* response. However, further research is required to elucidate a functional relationship.

In a parallel study, we explored the efficacy of IL-4 as an adjuvant in DC-mediated vaccination as in the context of visceral leishmaniasis, IL-4 mediates protective immunity and has been shown to instruct successful chemotherapy and vaccination responses (80). Accordingly, IL-4Rα-deficient BMDCs, with reduced IL-12 and increased IL-10 secretion, failed to vaccinate BALB/c animals against acute leishmaniasis, while IL-4-sufficient BMDCs producing increased IL-12 and reduced IL-10 successfully immunized BALB/c mice against infection (115). These observations unveiled yet another paradigm, suggesting that *Leishmania* vaccines should potentially incorporate IL-4 as an adjuvant, rather than IL-12, to induce protective IFN-γ responses.

## IL-4Rα Signaling on Specific Adaptive Immune Cells in CL in Mice

### IL-4Rα-Responsive CD4^+^ Th Cells in Murine CL

**Figure 8:**
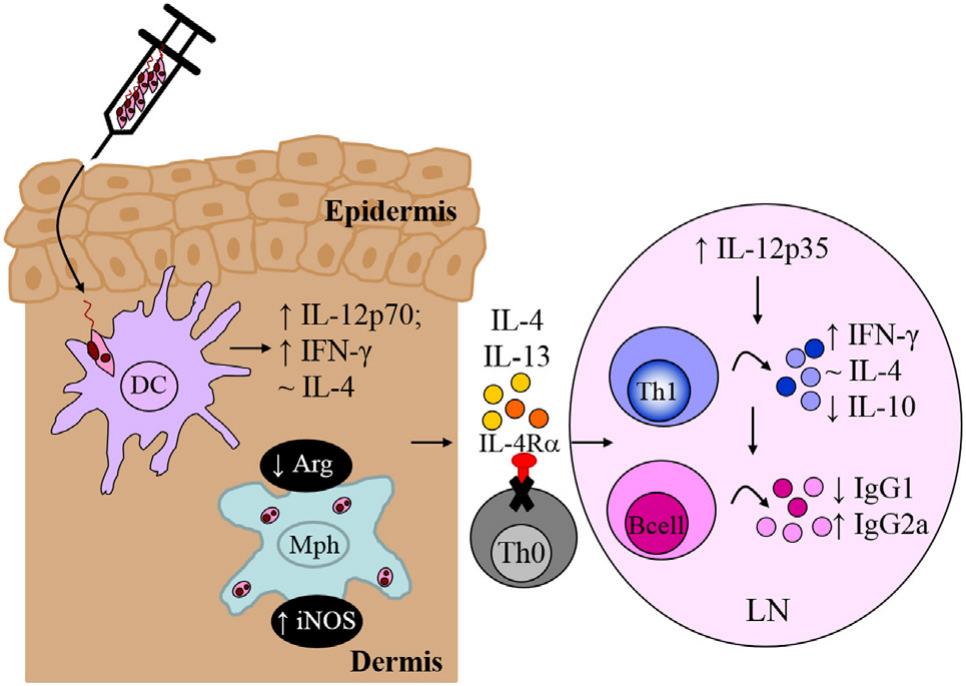
CD4^+^ T cell-specific interleukin-4 receptor alpha (IL-4Rα)-deficient mice develop a polarized Th1/type I immune response. In the absence of interleukin-4 (IL-4)-responsive T cells, normally susceptible BALB/c mice developed a healing phenotype upon experimental infection in the footpad, characterized by increased IFN-γ and IgG2a and concomitantly reduced IL-10 and IgG1 compared to littermate BALB/c mice. Interestingly, CD4^+^ T cell-specific IL-4Rα-deficient mice maintained equivalent IL-4 production to control littermate BALB/c mice. Illustration prepared from Radwanska et al. (10). DC, dendritic cells; Mph, macrophage; LN, lymph node; Th0, naive T cell; Th1, T helper 1.

Recognition of *Leishmania*-specific molecular patterns by the PRRs of early innate immune cells provides the necessary signals that determine the choice of effector response in adaptive immunity, mediated primarily by T and B lymphocytes. Indeed, the central role of T cells during *L. major* infection was established early on (116); however, the contradictory roles of IL-4 (7, 91) questioned whether IL-4 counter-regulated a protective Th1 response to promote susceptibility to infection. In an attempt to reconcile these observations, we focused on specific abrogation of IL-4-responsive CD4^+^ T cells by deletion of the IL-4Rα subunit in BALB/c mice (Lck^cre^IL-4Rα^−/lox^). In contrast to littermate control BALB/c mice, which developed ulcerating, necrotic lesions following infection with *L. major* LV39 and IL81, lack of IL-4-responsive CD4^+^ T cells led to a healing phenotype similar to that of the resistant C57BL/6 mice (10). Resistance to *L. major* in Lck^cre^IL-4Rα^−/lox^ BALB/c mice correlated with early *il-12p35* mRNA transcription leading to increased IFN-γ production, elevated iNOS expression and enhanced memory responses, similar to C57BL/6 mice (Figure 8). In contrast to global IL-4Rα^−/−^ mice, Lck^cre^IL-4Rα^−/lox^ BALB/c mice maintained chronic control of *L. major* infection. Collectively, the data demonstrated that CD4^+^ T cell-specific IL-4Rα-mediated signaling drives susceptibility to *L. major* infection, altogether highlighting a protective role for IL-4/IL-13 signaling on non-CD4^+^ T cells in *L. major*-infected BALB/c mice. Follow-up studies further demonstrated that iLck^cre^IL-4Rα^−/lox^ BALB/c mice, with IL-4Rα deficiency on all T cell populations (CD4^+^, CD8^+^, natural killer T, and γδ^+^ T cells) were also able to resolve lesion development, correlating with reduced parasite replication, IFN-γ-mediated protective delayed type hypersensitivity responses and downregulated *L. major*-specific IgG1, similar to Lck^cre^IL-4Rα^−/lox^ and C57BL/6 mice (Figure 8). As expected, the non-healing littermate BALB/c control mice developed severe footpad swelling and increased parasite burdens (81). Taken together, these studies concluded that absence of IL-4Rα-responsive non-CD4^+^, in addition to CD4^+^ T cells, does not further affect transformation of BALB/c mice to a healer phenotype.

### CD4^+^ Th17 and Tregs in CL in Relation to IL-4 and IL-4Rα Signaling

Apart from the Th1 and Th2 lineage of CD4^+^ T cells, naive CD4^+^ T cells may differentiate into additional lineages including CD4^+^ IL-17-producing cells (Th17) and Tregs. Th17 cells are characterized by the production of IL-17 and require retinoic acid-related orphan receptor gamma t, in addition to STAT3 and IL-23, for differentiation and maintenance (117). Various experimental models have linked the combined activity of Th17 cells, IL-17 and neutrophils to the pathogenesis of CL (118–120). A recent study indicated a link between IL-4 and the commitment of Th17 cells *via* the activation of IL-23 (121). In a model of cell-mediated inflammation, the authors demonstrated that IL-4 abrogates Th17 cells by selectively silencing IL-23 in APCs (121). Concurrently, IL-4 completely abrogates IL-23 to induce the IL-12-producing capacity of DCs. Altogether, the role of IL-4 and IL-4Rα signaling on this lineage of cells in CL would be an exciting avenue to investigate considering that in the study above, IL-12-dependent Th1 responses remained unaltered upon IL-4-mediated IL-23/Th17 silencing (121), and as already established, the former is needed for host protection to CL.

The ability for the host to maintain long-term immunity against *Leishmania* infection suggests that the parasite persists in an immune privileged site by a balance between CD4^+^ effector and Tregs that downregulate parasite-specific immunity (122). Differentiation of Tregs requires activation of forkhead box protein 3 (Foxp3) leading to the production of IL-10, TGF-β, and IL-4 (117, 123). In susceptible BALB/c mice, Tregs play a significant disease controlling role by regulating the biased Th2 response (ideally they suppress excessive Th2 responses) since the absence of CD4^+^CD25^+^ Treg cells dramatically increases IL-4 levels and exacerbates *L. major* infection (124, 125). In resistant C57BL/6 mice, CD4^+^CD25^+^ Tregs control protective Th1 responses by an IL-10-dependent mechanism mediating parasite persistence and latent infection (74). Of considerable interest, however, Pillemer et al. revealed that IL-4 signaling *via* the IL-4Rα–STAT6 axis was required to maintain Foxp3 expression in Tregs and promote their proliferation (123). Thus, while global depletion of Treg cells yielded informative and differing results in cutaneous disease, it provided little insight into the underlying mechanisms by which Tregs suppress or enhance Th2 responses *via* the IL-4/IL-4Rα axis (and the corresponding effects on Th1 immunity). The identification of Foxp3 as the crucial inducer of Tregs and our Cre/*lox*P technology enables us to provide further insight into this field. Consequently, we have generated BALB/c mice with a cell-specific deletion of the IL-4Rα chain on Tregs under control of the *foxp3* locus. Experimental studies in murine models are currently underway to elucidate the contribution of IL-4-signaling on Tregs in the infectious process caused by *L. major*.

### IL-4Rα-Responsive B Cells in Murine CL

Apart from CD4^+^ T cells, independent researchers began to unravel the contribution of B lymphocytes in host protection or susceptibility to CL. Initial proof-of-concept studies provided evidence that B cells may play a role in susceptibility to infection with *L. major* (126, 127). Nevertheless, the use of mice genetically deficient for B cells (μMT or J_H_D) demonstrated that B lymphocytes play a limited role in Th2-mediated susceptibility to *Leishmania* infection, as the absence of B cells in BALB/c mice did not critically alter disease outcome (128, 129). This conclusion changed dramatically when it became evident that B cells could also secrete cytokines that potentially modulate both pathologic or protective functions, independent of their antibody-secreting and antigen-presenting functions (130). This was further supported by several lines of evidence showing that B cells assist in regulating the quality and quantity of both primary and memory CD4^+^ Th cell responses (130). However, up until this point, the contribution of cytokine-producing B cells in the context of inflammation, infection, and autoimmunity, which were conventionally dependent on CD4^+^ T cells, was an unexplored aspect. A hallmark study employing both *in vitro* and *in vivo* methods characterized the subdivision of B cells into distinct cytokine-producing “effector” subsets. Defined effector B cells producing cytokines such as IFN-γ, IL-12p40, TNF-α, and IL-6 were termed B effector 1 (Be1) cells, whereas Be2 B cells secreted copious amounts of IL-2, IL-4, and IL-13 (23). The profiles of Be1 and Be2 cells closely resembled that of CD4^+^ Th1 and Th2 cells, which altogether led to a renaissance in this field of B cell biology. To date, cytokine-producing B cells have been reported in various models of parasitic and bacterial infection (23, 131, 132) and autoimmune diseases (133, 134). Collectively, the evidence provided by these studies were inclined to suggest that, in response to antigen, cytokine-producing B cells might function to initiate and/or maintain the magnitude and quality of CD4^+^ Th1/Th2-dependent immune responses.

**Figure 9:**
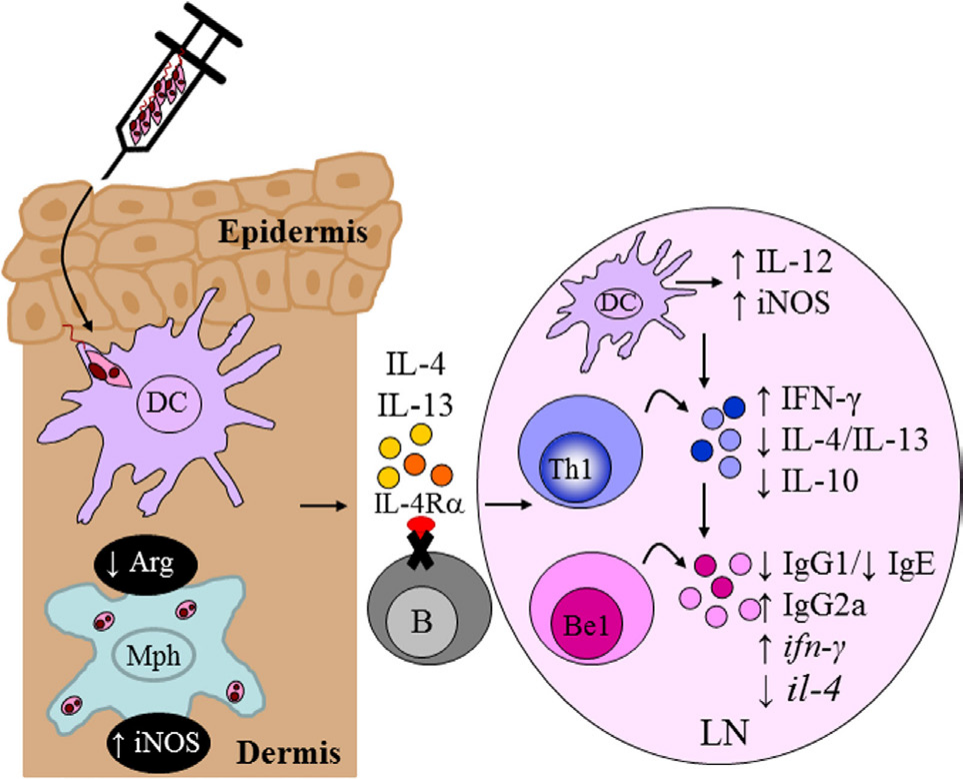
Absence of interleukin-4 (IL-4)-responsive B cells transforms non-healer BALB/c mice to a healer phenotype. Mb1^cre^IL-4Rα^−/lox^ mice developed an IL-12-induced protective Th1/type I immune response compared to littermate control BALB/c mice following *Leishmania major* LV39 and IL81 infection in the footpad. IL-4Rα-deficient B cells secreted increased *ifn-*γ and reduced *il-4* transcripts, as early as day 1 postinfection, resembling a B effector 1 phenotype in comparison to IL-4Rα-sufficient B cells. This in turn led to improved macrophage leishmanicidal functions and control of parasite replication at the site of infection. Illustration prepared from Hurdayal et al. (135). DC, dendritic cells; Mph, macrophage; LN, lymph node; B, B cells; Be1, B effector 1 B cells; Th1, T helper 1.

Of interest to our team, it was reported that the differentiation of naive B cells into IL-4-expressing Be2 cells was critically dependent upon Th2 signals and the IL-4/IL-4Rα signaling pathway (24). This led to the generation of a novel mouse strain lacking IL-4Rα expression specifically on B cells, mb1^cre^IL-4Rα^−/lox^ BALB/c mice, generated by cre/*lox*P under control of the *mb1* locus (56). As murine lymphocytes are not responsive to IL-13, this mouse model provided an invaluable tool to investigate a role for IL-4-responsive B cells during infection with *L. major*. Infection studies with *L. major* LV39 and IL-81 in B cell-specific IL-4Rα-deficient BALB/c mice revealed a beneficial role for IL-4Rα-unresponsive B cells in host-protective immunity and concomitantly, a detrimental role for IL-4Rα-responsive B cells in the non-healing response to *L. major* (135). In the absence of IL-4 signaling on B cells, BALB/c mice effectively controlled progression of lesion development and parasite replication as a consequence of enhanced Th1/type 1 immunity and improved leishmanicidal effector functions (Figure 9), resembling a similar phenotype to CD4^+^ T cell-specific IL-4Rα-deficient BALB/c (10). Mechanistic studies further revealed that the healing phenotype in mb1^cre^IL-4Rα^−/lox^ BALB/c mice was due to reduced *il-4* and increased *ifn-*γ transcripts in IL-4Rα-unresponsive B cells as early as Day 1 postinfection. Furthermore, mixed-bone marrow chimeras were instrumental in confirming that IL-4-producing B cells are crucial in driving the non-healing susceptible type 2 immune response characteristic of BALB/c mice during *L. major* infection (135). Recent evidence has indicated innate-like activation of B cells following recognition of foreign antigens (136, 137), *via* B cell receptor-independent mechanisms, leading to early B cell cytokine secretion. The efficient antigen-presenting function of B cells (138, 139) allows the cell to present the antigen it acquires and in lieu of its cytokine secretion, induce paracrine activation of neighboring cells, such as CD4^+^ T cells. These reports and our current data appear to suggest a model in which early IL-4Rα-responsive B cells producing IL-4 after infection are capable of influencing early Th polarization toward detrimental Th2 responses that drive *L. major*-induced CL (135).

## Conclusion

Collectively, the development of conditional mice and generation of important immune cell-type-specific IL-4Rα-deficient mouse models have been critical in our understanding of Th2/type 2-mediated mechanisms in innate and adaptive immune cells during disease with the causative agent of CL, *L. major*. Our data show that the hierarchical importance of target cells interacting with the IL-4Rα and its ligands (IL-4 and IL-13) is a dynamic interaction, largely influenced by cytokines secreted in the vicinity of target and non-target cells, heterogenous CD4^+^ T cell populations, autocrine versus paracrine signaling, the *Leishmania* species/strain initiating infection, and the timing of the immune response.

This is especially relevant when considering the full spectrum of human leishmaniasis and strain-specific roles of the IL-4Rα chain and its ligands. Visceral leishmaniasis, initiated by intravenous injection of *Leishmania donovani* amastigotes in mouse models, is quite idiosyncratic in this regard. Similar to *L. major* infection in BALB/c mice, protective immunity against *L. donovani* is dependent on IL-12-induced-IFN-γ production for classical activation of macrophages and NO-induced killing of intracellular parasites (140). However, early studies in both humans and mice demonstrated that control of visceral disease was independent of the differential production of Th1 and Th2-derived cytokines (141). Thus, the overall evidence suggested that the Th2 response did not counteract immunological control of visceral leishmaniasis (142, 143).

On the contrary, the introduction of gene-deficient mice revealed surprisingly protective roles for IL-4, IL-13 and IL-4Rα signaling, during primary *L. donovani* infection (144–146). In addition, the IL-4/IL-13 signaling cascade was reported to play a significant role in successful drug treatment with sodium stibogluconate (80, 145) and augmenting vaccination responses (147). These reports added immense value to our understanding of the Th2 response in remarkably, control of visceral leishmaniasis, improved chemotherapy and vaccination. However, it could not tell us which IL-4/IL-13-signaling cells were important in mediating protective immunity, in lieu of the global gene deficiency in these models. Consequently, current research within our team is aimed at unraveling IL-4 and IL-13 signaling on specific target immune cells during visceral leishmaniasis using our cell-type-specific IL-4Rα-deficient mouse models. Our initial studies have indicated that control of primary *L. donovani* infection, granuloma maturation, and chemotherapeutic efficacy is independent of IL-4Rα-responsive macrophages and neutrophils in BALB/c mice (144). This raises intriguing questions regarding the modes of action of IL-4 and IL-13 on other cellular targets such as CD4^+^ T cells, Treg cells, DCs, and B cells, which are currently under investigation.

## Author Contributions

RH and FB contributed equally to the design and writing of the present review.

## Conflict of Interest Statement

The authors declare that the research was conducted in the absence of any commercial or financial relationships that could be construed as a potential conflict of interest.
